# Comparative cost-effectiveness of sintilimab, toripalimab, and camrelizumab in first-line therapy for advanced non-squamous non-small cell lung cancer

**DOI:** 10.3389/fpubh.2025.1754642

**Published:** 2026-01-14

**Authors:** Jie Huang, Wei Zhang, Bangyu Zhang

**Affiliations:** ^1^Office of Clinical Research Management, Shanghai Pulmonary Hospital, School of Medicine, Tongji University, Shanghai, China; ^2^Department of Pharmacy, Shanghai Pulmonary Hospital, School of Medicine, Tongji University, Shanghai, China

**Keywords:** Chinese national medical insurance system, cost-effectiveness analysis, Markov model, non-small cell lung cancer, PD-1 inhibitors

## Abstract

**Background:**

Several PD-1 inhibitors used in first-line treatment of advanced non-squamous non–small cell lung cancer in China, including sintilimab, toripalimab and camrelizumab, have demonstrated significant survival benefits in phase III trials. However, their comparative cost-effectiveness within the Chinese national medical insurance system remains unclear.

**Methods:**

A Markov model with progression-free, progressive disease and death states was developed from the Chinese national medical insurance system payer perspective. Clinical efficacy inputs were obtained from three China-based phase III randomized trials. Individual patient data were reconstructed from published Kaplan–Meier curves using the Guyot method, and parametric survival models were fitted for extrapolation. Costs included drug acquisition, administration, adverse event management and post-progression therapy. Outcomes were total costs, quality-adjusted life-years (QALYs) and incremental cost-effectiveness ratios (ICERs). One-way and probabilistic sensitivity analyses were conducted.

**Results:**

Sintilimab incurred the lowest cost (230,813 CNY) and generated 1.1 QALYs. Toripalimab yielded the same QALYs at a higher cost and was strictly dominated. Camrelizumab produced the highest QALYs (1.2) at a total cost of 253,056 CNY. Compared with sintilimab, camrelizumab had an ICER of 164,983 CNY/QALY, below the willingness-to-pay threshold of 287,247 CNY/QALY. Sensitivity analyses confirmed the robustness of these findings.

**Conclusion:**

Among the three domestic PD-1 inhibitors evaluated, camrelizumab is the most cost-effective first-line treatment option for advanced non-squamous NSCLC in China.

## Introduction

Lung cancer is one of the most common malignancies in China and continues to pose a substantial threat to public health. According to the most recent data from the National Cancer Center (NCC), the crude incidence and mortality rates of lung cancer in 2022 reached 75.13 and 51.94 per 100,000 population, respectively, both of which were highest among all cancers nationwide (1). Non-small cell lung cancer (NSCLC) accounts for the vast majority of all lung cancer cases, with non-squamous histology representing the predominant subtype (2). Studies indicate that more than half of lung cancer patients in China are already at an advanced stage at the time of diagnosis, thereby losing the opportunity to undergo curative surgery (2–5). With the rapid advances in immuno-oncology over the past few years, systemic treatment strategies for NSCLC have made remarkable progress. In addition to conventional platinum-based doublet chemotherapy, programmed cell death-1 (PD-1) inhibitors have quickly become a central component of standard care. Multiple international and Chinese guidelines now recommend PD-1 inhibitors combined with pemetrexed and a platinum agent as a first-line standard regimen for patients with advanced NSCLC, especially those with non-squamous histology (6–8).

Several domestically developed PD-1 inhibitors have demonstrated robust and consistent clinical benefits in randomized controlled trials (RCTs) conducted among Chinese patients. As one of the earliest PD-1 inhibitors approved for NSCLC in China, toripalimab combined with chemotherapy significantly prolonged progression-free survival (PFS; median, 9.7 months), while overall survival (OS) had not yet been reached at the time of the primary analysis (median OS: not reached), and grade ≥3 adverse events were reported in 78.6% of patients (9). Similarly, camrelizumab in combination with pemetrexed and platinum chemotherapy achieved a marked improvement in PFS (median PFS: 11.0 months) and significantly prolonged overall survival (median OS: 27.1 months), with grade ≥3 adverse events observed in 70.7% of patients (10). In another large China-based phase III trial, sintilimab combined with pemetrexed and platinum chemotherapy resulted in substantial gains in PFS (median PFS: 8.9 months), while median OS was not reached at the time of reporting (median OS: not reached), and grade ≥3 adverse events occurred in 61.7% of patients (11). Across these phase III trials, the PD-1–based chemo-immunotherapy regimens demonstrated generally comparable efficacy profiles, while differences in the incidence and spectrum of treatment-related adverse events were observed among the regimens. Taken together, these phase 3 trial findings indicate that the major domestically developed PD-1 inhibitors in China have all demonstrated clear therapeutic efficacy in multiple phase III RCTs involving patients with advanced non-squamous NSCLC.

However, as China rapidly transitions into an aging society, the economic burden of lung cancer on the healthcare system has grown substantially, with drug expenditures being one of the major contributors to overall lung cancer–related medical costs (12). Although several domestically developed PD-1 inhibitors have been approved in recent years—greatly expanding the range of first-line treatment options and offering patients more opportunities for clinical benefit—the relative cost-effectiveness of these PD-1–based chemo-immunotherapy regimens in the Chinese healthcare context remains uncertain. In light of increasing pressure on the Chinese national medical insurance system, there is an urgent need for pharmacoeconomic evaluations based on Chinese clinical trial evidence and real-world cost data to compare the cost-effectiveness of different first-line immunotherapy strategies. Such analyses are essential to inform clinical decision-making and guide healthcare policy development.

To address this critical evidence gap and to comprehensively evaluate the cost-effectiveness of the major domestically developed PD-1 inhibitors in first-line therapy, rigorous pharmacoeconomic analyses based on Chinese clinical and cost data are needed to support clinical decision-making and policy planning, particularly in light of differences in drug acquisition costs and real-world market uptake despite similar reimbursement status. Therefore, this study aims to compare the cost-effectiveness of three domestic PD-1 inhibitors (sintilimab, toripalimab and camrelizumab) as first-line treatments for patients with advanced non-squamous non–small cell lung cancer in China.

## Methods

### Clinical data sources

This study was conducted from the perspective of the Chinese national medical insurance system payer. To ensure that the clinical inputs reflected treatment outcomes in the Chinese population, efficacy data were obtained from three phase III, randomized, controlled, multicenter clinical trials conducted exclusively in China (Table 1): CameL study (camrelizumab) (10), ORIENT-11 study (sintilimab) (11), and CHOICE-01 study (toripalimab) (9). All three trials evaluated pemetrexed combined with a platinum agent as the treatment regimen and compared it with chemotherapy alone, allowing for strong clinical comparability among the three PD-1 plus chemotherapy strategies. Data on progression-free survival (PFS), overall survival (OS), and treatment-related adverse events (AEs) required for the model were systematically extracted from the corresponding trial publications to ensure accuracy and consistency of input parameters. For the CHOICE-01 trial, data were extracted exclusively from the non-squamous NSCLC subgroup.

**Table 1:** Model parameters and distribution.

Parameters	Base value	Low	High	Distribution	Source
Cycle-based treatment costs					
Cost of camrelizumab	3,558	2,519	3, 558	Gamma	Local
Cost of sintilimab	3,558	2,519	3, 558	Gamma	Local
Cost of toripalimab	3,636	2,727	3,636	Gamma	Local
Cost of pemetrexed	833	625	1,041	Gamma	Local
Cost of carboplatin	364	273	455	Gamma	Local
Cost of drug administration	6,348	4,761	7,935	Gamma	Local
Cost of subsequent second-line therapy	2,792	2,094	3,490	Gamma	(16)
Adverse event cost					
Neutrophil count decreased	818	363	2,545	Gamma	(15)
White blood cell count decreased	818	363	2,545	Gamma	(15)
Platelet count decreased	10,707	8,818	12,597	Gamma	(15)
Anemia	987	759	1,138	Gamma	(15)
Utility values					
Utility of PFS	0.804	0.536	0.883	Beta	(14)
Utility of PD	0.321	0.05	0.473	Beta	(14)
Discount rate (%)	5	0	8		(17)

This table summarizes the cost, utility and clinical parameters applied in the Markov model. All cost inputs are presented in Chinese yuan (CNY) and include cycle-based treatment costs, drug administration, and subsequent second-line therapy. Adverse event costs reflect grade 3 or higher events incorporated into the analysis. Utility values for progression-free and progressive disease health states were derived from published studies in patients with advanced non–small cell lung cancer. Local indicates data collected by the authors in this study. Parameter uncertainty was modeled using gamma distributions for cost variables and beta distributions for utility values. The lower and upper bounds represent the ranges used in one-way sensitivity analyses.

The other baseline characteristics and clinical outcomes of the CameL, ORIENT-11, and CHOICE-01 trials are summarized in Supplementary Table S1.

### Markov model structure

**Figure 1:**
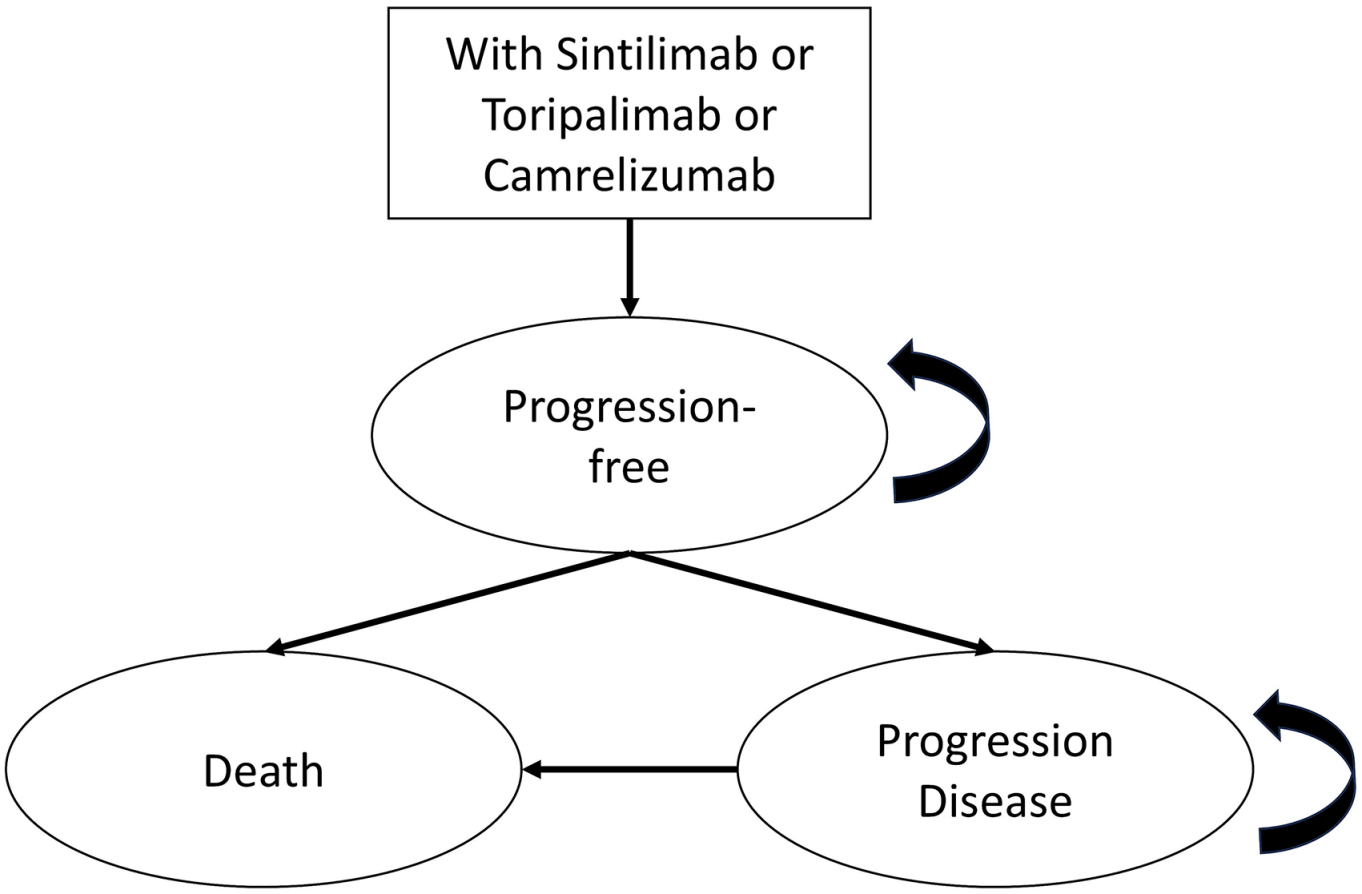
Markov model structure. This figure illustrates the three-state Markov model used to evaluate first-line treatment with sintilimab, toripalimab, or camrelizumab. All patients enter the model in the progression-free state and may transition each cycle to progressive disease or death. Death is modeled as an absorbing state. The cycle length was set at 3 weeks, and transitions reflect survival outcomes derived from parametric models extrapolated from phase III clinical trials.

A Markov model with three mutually exclusive health states was developed for this study. The health states included progression-free (PF), progressive disease (PD) and death. All patients entered the model in the PF state, and death was defined as an absorbing state. Based on background mortality and transition probabilities derived from clinical trials, patients in the PF state could transition to either PD or death. Patients in the PD state could remain in PD or transition to death (Figure 1). Given the irreversible nature of advanced NSCLC, backward transitions between health states were not permitted.

The cycle length of the model was set at 3 weeks to reflect the clinical dosing and follow-up schedule. A lifetime horizon was adopted to capture long-term survival outcomes. The model was constructed and analyzed using TreeAge Pro 2022 (TreeAge Software Inc., Williamstown, MA, United States). Total costs and quality-adjusted life-years (QALYs) were estimated for each treatment strategy, and the incremental cost-effectiveness ratio (ICER) was calculated as the primary outcome measure for evaluating cost-effectiveness.

### Transition probability estimation

Transition probabilities between health states are key parameters in a Markov model and have a critical influence on cost and effectiveness outcomes. Because individual patient data (IPD) are typically not available from most phase III randomized controlled trials (RCTs), this study applied the reconstruction method proposed by Guyot et al. to approximate IPD from published Kaplan–Meier curves (13).

First, PFS and OS KM curves reported in the RCT publications were digitized using the software automeris.io (version 5, https://automeris.io/). Second, the digitized KM data were used to reconstruct pseudo-IPD following the Guyot approach. Third, several commonly used parametric survival distributions, including exponential, Weibull, log-normal, log-logistic and Gompertz, were fitted to the reconstructed IPD. The optimal distribution for extrapolation was selected based on the Akaike information criterion (AIC) and the Bayesian information criterion (BIC).

Finally, cycle-specific transition probabilities for disease progression and death were derived from the fitted survival functions. These transition probabilities were applied separately for each treatment group to reflect differences in disease trajectories across the PD-1 plus chemotherapy regimens.

### Utility inputs

Health state utility values were obtained from a published health-related quality-of-life study, with 0 representing death and 1 representing perfect health. This study adopted utility estimates specifically reported for Chinese patients with advanced non-squamous non–small cell lung cancer (14). The utility value for the progression-free (PFS) state was 0.804, and the utility value for the progressive disease (PD) state was 0.321 (Table 1).

### Cost inputs

This study was conducted from the perspective of the Chinese national medical insurance system payer and therefore included only direct medical costs. The cost components considered in the analysis are listed in Table 1.

Drug costs and disease management costs were obtained directly from actual procurement prices and medical service fees at a specialty hospital in China, collected by the study team to represent the cost structure of Chinese public hospitals. Costs for managing adverse events were derived from published Chinese cost estimates (15), while the incidence of AEs was extracted from the major grade 3 or higher adverse events reported in the three phase III RCTs (9–11). These AE incidence rates were used to calculate the weighted average cost of AE management incorporated into the model (Table 2).

**Table 2:** Incidence rate of treatment-related adverse events.

Adverse events	Camrelizumab (10)	Sintilimab (11)	Toripalimab (9)
Neutrophil count decreased	39.5%	36.5%	55.5%
White blood cell count decreased	20%	14.7%	35.7%
Platelet count decreased	16.6%	12%	17.2%
Anemia	20%	15%	29.9%

This table presents the incidence rates of selected grade 3 or higher treatment-related adverse events reported in the phase III clinical trials of camrelizumab, sintilimab and toripalimab. These adverse events were included in the cost calculations for adverse event management in the Markov model. Values are expressed as percentages of patients in each treatment group.

In addition, for patients who progressed after first-line treatment, we assumed that docetaxel was used as the subsequent second-line chemotherapy (16). The cost of docetaxel and its administration was included in the model to reflect post-progression treatment expenses. In accordance with the China Guidelines for Pharmacoeconomic Evaluations, both costs and utilities were discounted at an annual rate of 5%, and a willingness-to-pay (WTP) threshold of three times the gross domestic product (GDP) per capita was used as a reference (17). All costs were reported in Chinese yuan (CNY). Currency conversions, if needed, were adjusted using the 2025 exchange rates.

### Sensitivity analyses

To evaluate the robustness of the model outcomes, both one-way sensitivity analysis and probabilistic sensitivity analysis (PSA) were conducted. First, the one-way sensitivity analysis assessed the impact of varying key model parameters within predefined ranges on the incremental cost-effectiveness ratio (ICER). Results were presented using tornado diagrams. For parameter ranges, only price reductions were applied to sintilimab, toripalimab and camrelizumab because all three PD-1 inhibitors are listed in the Chinese National Reimbursement Drug List and price increases are unlikely under the current policy environment. For other parameters without available range estimates, a variation of ±25 percent was applied.

Second, the probabilistic sensitivity analysis incorporated simultaneous uncertainty across all model parameters through 10,000 Monte Carlo simulations. Gamma distributions were assigned to cost parameters, and Beta distributions were applied to utility values and transition probabilities between health states (Table 1). Results were presented as cost-effectiveness acceptability curves (CEACs) to illustrate the probability of each treatment strategy being cost-effective at different WTP thresholds.

## Results

### Basic analysis

In the basic analysis (Table 3), the three treatment strategies showed notable differences in total costs and health outcomes measured in quality-adjusted life-years (QALYs). Sintilimab had the lowest total cost at 230,813 CNY and generated 1.1 QALYs, making it the least costly option among the three regimens. Toripalimab incurred a total cost of 243,082 CNY and produced the same health benefit as sintilimab (1.1 QALYs). Because toripalimab was more costly and offered no additional health benefit, it was identified as a strictly dominated strategy and excluded from the subsequent incremental cost-effectiveness analysis. In contrast, camrelizumab resulted in a total cost of 253,056 CNY and generated 1.2 QALYs, which was 0.1 QALYs higher than sintilimab. Using sintilimab as the reference, camrelizumab had an incremental cost of 22,243 CNY, yielding an incremental cost-effectiveness ratio (ICER) of 164,983 CNY per QALY gained. Using three times China’s 2024 per capita GDP (95,749 CNY) as the WTP threshold, equivalent to 287,247 CNY per QALY, the ICER for camrelizumab was well below this threshold. These findings indicate that camrelizumab is the most cost-effective option among the three domestic PD-1 inhibitors within the Chinese healthcare system.

**Table 3:** Basic analysis.

Treatment	Cost	Δ Cost	QALYs	Δ QALYs	ICER vs. sintilimab
Sintilimab	230,813	Reference	1.1	Reference	Reference
Toripalimab (dominated)	243,082	12,269	1.1	0.1	195,661
Camrelizumab	253,056	22,243	1.2	0.1	164,983

This table summarizes the total costs, quality-adjusted life-years (QALYs), incremental outcomes and incremental cost-effectiveness ratios (ICERs) for the three first-line treatment strategies. All costs are reported in Chinese yuan (CNY). Sintilimab had the lowest total cost, whereas toripalimab generated identical QALYs at a higher cost and was therefore considered a dominated option. Camrelizumab yielded the highest QALYs with an ICER below the willingness-to-pay threshold, indicating favorable cost-effectiveness in the base-case analysis.

### Sensitivity analyses

#### One-way sensitivity analysis

**Figure 2:**
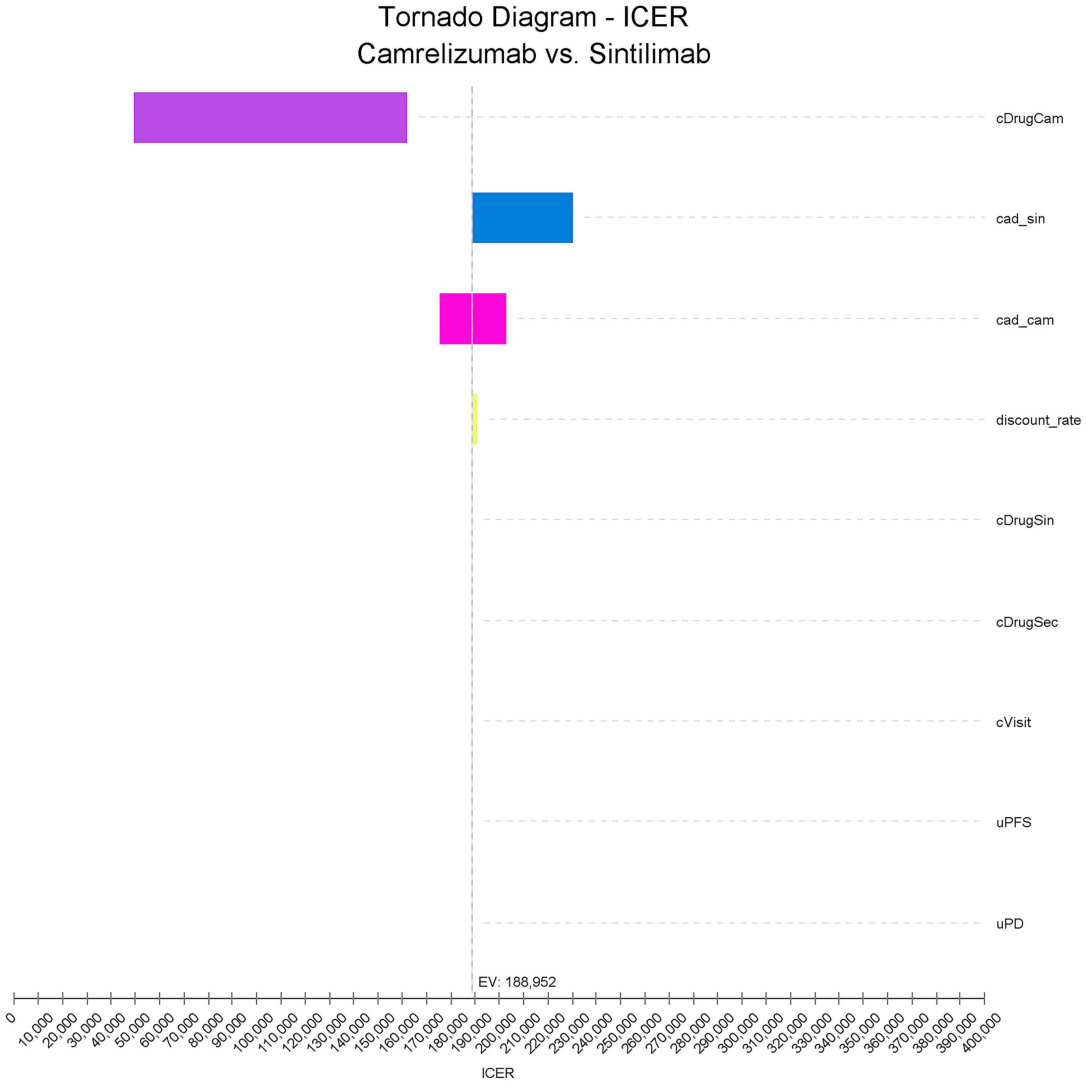
One-way sensitivity analysis. This tornado diagram illustrates the influence of key parameters on the incremental cost-effectiveness ratio (ICER, CNY/QALY) of camrelizumab compared with sintilimab in the base-case analysis. Each bar represents the change in ICER when the specified parameter is varied across its predefined range. The cost of camrelizumab (cDrugCam) exerted the largest impact on the ICER, followed by the adverse event management cost for sintilimab (cad_sin) and camrelizumab (cad_cam). Other parameters, including the discount rate, the drug cost of sintilimab (cDrugSin), the cost of second-line therapy (cDrugSec), visit and administration cost (cVisit), and utility values for progression-free (uPFS) and progressive disease (uPD) states, produced minimal variation in the ICER, supporting the robustness of the model results.

The one-way sensitivity analysis evaluated how variations in individual parameters affected the incremental cost-effectiveness ratio (ICER) of camrelizumab compared with sintilimab (Figure 2). The results indicated that the drug cost of camrelizumab (cDrugCam) was the most influential parameter, producing the largest fluctuation in the ICER within its plausible range. This finding shows that the price of camrelizumab plays a dominant role in determining the overall cost-effectiveness conclusion. The post-progression adverse event management cost for sintilimab (cad_sin) also had a moderate impact on the ICER. Changes in this parameter led to noticeable increases or decreases in the ICER, indicating that the cost of managing adverse events contributes to model sensitivity. The adverse event management cost associated with camrelizumab (cad_cam) produced a smaller but still observable effect on the ICER, although its influence was limited compared with drug acquisition costs. In contrast, variations in the discount rate, the drug cost of sintilimab (cDrugSin), the cost of second-line therapy (cDrugSec), visit and administration costs (cVisit), and the utility values for the progression-free and progressive disease states (uPFS and uPD) had only minimal effects on the ICER. Even when varied within their defined ranges, these parameters produced only minor fluctuations that did not alter the overall model conclusions. Overall, the one-way sensitivity analysis indicates strong robustness of the model.

#### Probabilistic sensitivity analysis

**Figure 3:**
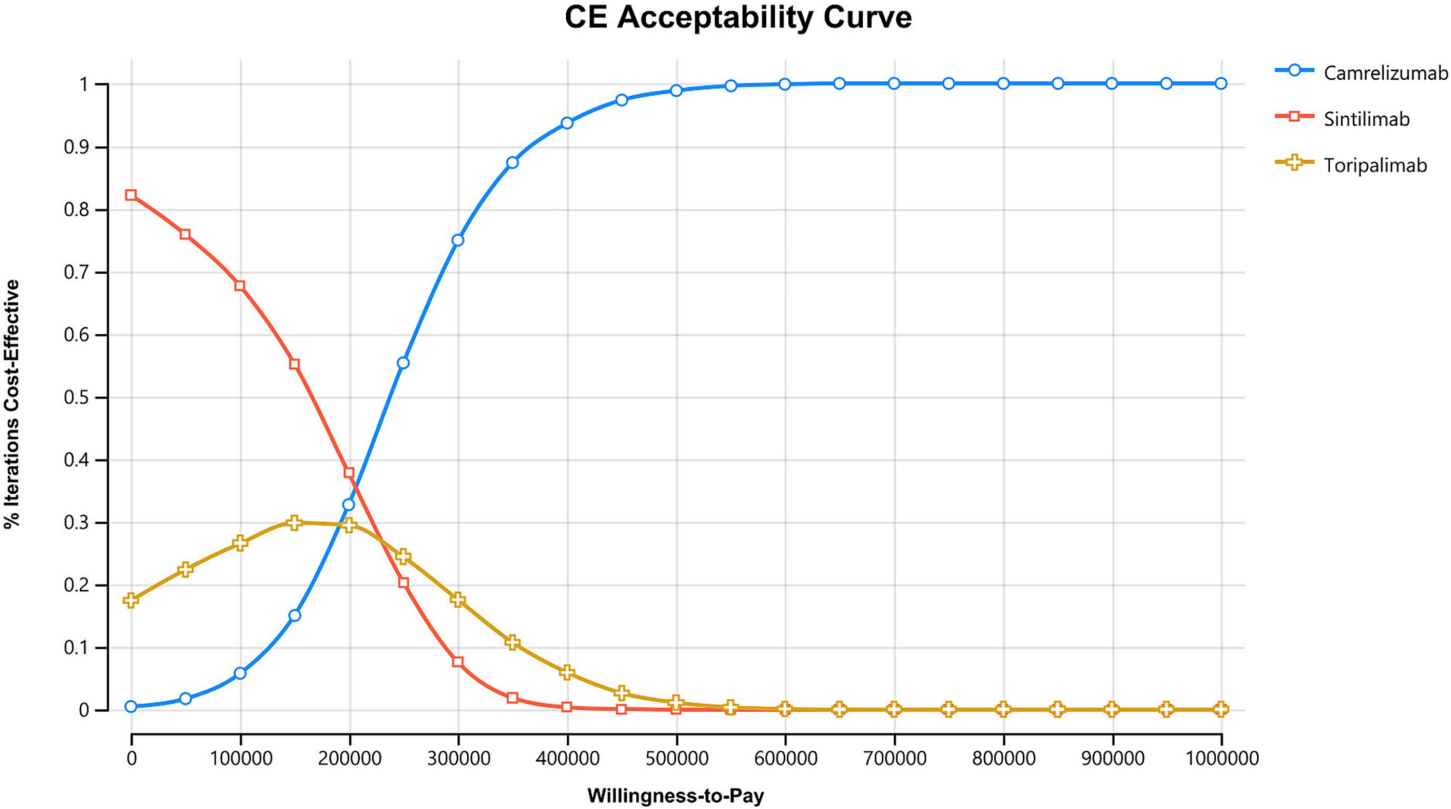
Cost-effectiveness acceptability curve (CEAC). This figure illustrates the probability that camrelizumab, sintilimab, or toripalimab is cost-effective across a range of willingness-to-pay (WTP) thresholds based on 10,000 Monte Carlo simulations. Sintilimab shows the highest probability of being cost-effective at lower WTP levels. As the WTP threshold increases, camrelizumab becomes increasingly favorable and shows the highest probability at higher WTP values. Toripalimab maintains a low probability of cost-effectiveness across the full range of thresholds.

Based on 10,000 Monte Carlo simulations, this study assessed the probability that each treatment strategy would be the most cost-effective option across a range of WTP thresholds (Figure 3). Using three times the 2024 per capita GDP of China (95,749 CNY), a WTP threshold of 287,247 CNY per QALY was applied as the primary reference. At lower WTP levels (less than 150,000 CNY per QALY), sintilimab demonstrated the highest probability of being cost-effective, with approximately 54 percent of simulations identifying it as the preferred strategy. As the WTP threshold increased, the advantage of sintilimab gradually diminished, and its cost-effectiveness curve intersected with that of camrelizumab at around 200,000 CNY per QALY. At the primary WTP threshold of 287,247 CNY per QALY, camrelizumab exhibited a markedly higher probability of being cost-effective, reaching approximately 70 percent. This indicates that camrelizumab outperformed both sintilimab and toripalimab in the majority of PSA simulations. As WTP values continued to rise, the probability that camrelizumab would be the most cost-effective option increased further, demonstrating a consistent and pronounced advantage.

## Discussion

This study evaluated the cost-effectiveness of three domestically developed PD-1 inhibitors, sintilimab, toripalimab and camrelizumab, for first-line treatment of advanced non-squamous NSCLC from the perspective of the Chinese national medical insurance system payer. All three immunotherapy regimens are included on the Chinese National Reimbursement Drug List and are widely used in clinical practice, making an economic comparison highly relevant for both policy and clinical decision-making. Based on the Markov model analysis and using three times China’s 2024 per capita GDP as the WTP threshold, camrelizumab emerged as the most cost-effective option. In probabilistic sensitivity analysis, the mean QALYs (95% uncertainty interval) were 1.1 (0.9–1.2) for sintilimab and 1.1 (1.0–1.2) for toripalimab. The substantial overlap in QALY distributions, combined with consistently higher total costs associated with toripalimab, supports its classification as a strictly dominated strategy. As a result, toripalimab was strictly dominated and showed no cost-effectiveness advantage under the conditions of this study.

Most previous cost-effectiveness analyses have compared a single PD-1 inhibitor combined with pemetrexed and platinum chemotherapy against chemotherapy alone (16, 18–23), and few studies have directly compared multiple major PD-1 inhibitors. Given the increasing use of domestically developed PD-1 inhibitors in China, evaluating the cost-effectiveness of several mainstream regimens simultaneously has important clinical and policy relevance. This study addresses an existing evidence gap in the literature. Findings from previous studies evaluating the cost-effectiveness of camrelizumab have not been consistent (16, 24, 25). The differences between earlier studies and the present analysis can be explained by several factors. First, the prices of PD-1 inhibitors and overall treatment costs in China vary annually and regionally depending on national reimbursement negotiations, procurement mechanisms and the level of medical institutions. By using real procurement prices from a specialty hospital in China, this study ensured that cost inputs closely reflected actual clinical practice, providing greater relevance and applicability compared with studies that relied on literature-derived or international pricing data. Second, the incidence of grade 3 or higher adverse events reported in phase III trials varies across PD-1 regimens. In China, the cost of managing adverse events, especially after disease progression, can account for a substantial portion of medical expenditures. As a result, heterogeneity in adverse event profiles can meaningfully affect ICER outcomes. The one-way sensitivity analysis in this study confirmed this pattern. Finally, this study reconstructed individual patient data using the Guyot method (13), applied several parametric survival distributions for extrapolation, and selected the best-fitting model based on the Akaike information criterion and the Bayesian information criterion. This approach improved the accuracy of long-term survival projections. Some earlier studies relied on simplified interpretations of Kaplan–Meier curves or used a single distribution for extrapolation, which may underestimate or overestimate the long-term benefits of PD-1 therapy and thereby shift ICER values (26, 27). In the probabilistic sensitivity analysis, survival uncertainty had a relatively small effect on the ICER, indicating that the transition probability estimates in this study were stable and robust.

A closer comparison of the three PD-1 inhibitors evaluated in this study indicates that the observed differences in cost-effectiveness can be attributed to three major drivers: drug acquisition costs and total treatment expenditures, the incidence and management costs of adverse events and the post-progression survival trajectories associated with each regimen. These factors jointly influence total costs and QALYs, ultimately determining the relative ICERs. First, camrelizumab demonstrated better progression-free survival and overall survival outcomes in clinical trials, resulting in the highest QALY gain in the model. Longer survival implies a longer duration of immunotherapy and additional subsequent medical spending, which increases its overall cost. However, the substantial incremental health benefit it provided kept its ICER well below commonly used WTP thresholds in China, making it the most cost-effective strategy overall. Second, although the health benefit of sintilimab was slightly lower than that of camrelizumab, its total cost was substantially lower. This allowed sintilimab to remain competitive at lower or moderate WTP levels. This finding highlights the critical role of drug acquisition costs in determining the cost-effectiveness of immunotherapy and is consistent with the one-way sensitivity analysis, in which drug price was identified as a principal driver. In contrast, toripalimab was more costly than sintilimab yet did not generate additional QALYs in the model. As a result, it offered no advantage in either cost or effectiveness and was strictly dominated. Neither its adverse event profile nor its post-progression survival trajectory was sufficient to offset its higher expenses, leading to markedly inferior cost-effectiveness compared with the other two PD-1 inhibitors.

From a health policy perspective, the findings of this study have important implications for reimbursement decision-making within the Chinese national medical insurance system. Although sintilimab, toripalimab, and camrelizumab are all included in the National Reimbursement Drug List following price negotiations, differences in long-term value remain when both clinical benefits and economic consequences are considered. Our results suggest that camrelizumab-based chemo-immunotherapy provides the greatest health gains and the highest probability of cost-effectiveness under commonly accepted WTP thresholds in China. Such evidence may support value-based resource allocation and inform future reimbursement adjustments, pricing negotiations, and clinical guideline development amid increasing budgetary pressure from an aging population and the rising burden of lung cancer.

There are several limitations to this study. First, the clinical effectiveness inputs were primarily derived from large randomized controlled trials, which are conducted under idealized conditions and may not fully represent real-world variations in treatment adherence, dosing frequency, comorbidity burden or subsequent therapy choices (28). Second, the three PD-1 regimens included in this analysis were not compared directly within the same clinical trial but were obtained from separate phase III studies. Although all included trials were conducted in China and evaluated similar first-line chemo-immunotherapy regimens, and reconstructed individual patient data together with multiple parametric survival models were applied to enhance comparability, this indirect comparison approach may still be subject to residual bias arising from differences in trial design, patient characteristics, follow-up duration, and post-progression treatment strategies (29). Furthermore, while our Markov model addresses the potential for logical inconsistencies (e.g., crossing survival curves) inherent in partitioned survival models (PSMs), it shares with PSMs the structural uncertainty associated with the choice of parametric distributions for long-term survival extrapolation. Third, the costs of post-progression treatments and adverse event management were sourced from published literature or hospital-level pricing data, which may introduce some uncertainty. Despite these limitations, the probabilistic sensitivity analysis showed that the main findings remained consistent across a wide range of parameter variations, supporting the robustness of the model results.

## Conclusion

This study compared the cost-effectiveness of three major domestically developed PD-1 inhibitors for first-line treatment of advanced non-squamous non–small cell lung cancer in China. The findings indicate that camrelizumab is the most cost-effective option in the current medical insurance reimbursement environment, while sintilimab remains an attractive alternative for scenarios with lower WTP thresholds, given its lower total cost. These results provide useful evidence to inform clinical treatment selection and support policy development within the Chinese healthcare system. Further real-world data are needed to validate the long-term economic value of PD-1 therapies in routine clinical practice in China.

## Data Availability

Publicly available datasets were analyzed in this study. This data can be found at: all data supporting this cost-effectiveness analysis are fully described within the manuscript. Clinical efficacy inputs were obtained from published phase III trials, while cost data were derived from both publicly available literature and real-world procurement information collected from a public hospital. All self-collected cost data have been explicitly reported in the manuscript, and no additional unpublished datasets were used.
