# Supplementary material for: Comparative cost-effectiveness of sintilimab, toripalimab, and camrelizumab in first-line therapy for advanced non-squamous non-small cell lung cancer
Source: Front Public Health. 2026 Jan 14;13:1754642. doi: 10.3389/fpubh.2025.1754642 (PMC12848809; doi:10.3389/fpubh.2025.1754642)
Supplement: Supplementary file 1 [file Table_1.docx]

**Supplementary**

**Table S1. Baseline characteristics and outcomes from the three trials (CameL, ORIENT-11, CHOICE-01)**

| **Characteristic** | **CameL (10)** | **ORIENT-11 (11)** | **CHOICE-01 (9)** |
| --- | --- | --- | --- |
| **PD-1 inhibitor** | Camrelizumab | Sintilimab | Toripalimab |
| **Region** | China | China | China |
| **Sample size** | 205 | 266 | 162 |
| **Eligible age** | 18-70 | 18-75 | 18-75 |
| **Median PFS (months)** | 11.0 (8.5-12.5) | 8.9 (7.1-11.3) | 9.7 (8.2-13.1) |
| **Median OS (months)** | 27.1 (21.9-31.5) | NR (NR,NR) | NR (NR,NR) |
| **Grade ≥3 AEs (%)** | 70.7% | 61.7% | 78.6% |
| **Note:** AE, adverse event; HR, hazard ratio; OS, overall survival; PD-1, programmed death 1; PFS, progression-free survival; NR, not reached. Data were extracted from the published primary analyses of the CameL, ORIENT-11, and CHOICE-01 phase III trials. For CHOICE-01, only the non-squamous NSCLC subgroup was included when available. Comparisons were descriptive and not intended as formal statistical comparisons. | | | |
